# A novel electronic patient-reported outcome delivery system to implement health-related quality of life measures in routine clinical care: An analysis of 5 years of experience

**DOI:** 10.3389/fdgth.2022.1074931

**Published:** 2023-01-09

**Authors:** Kathleen Tymms, Catherine O'Sullivan, Tegan Smith, Geoffrey Littlejohn, Tim Freeman, David Hoffman, Dana Segelov, Hedley Griffiths, Sabina Ciciriello, Peter Youssef, David Mathers, Claire T Deakin

**Affiliations:** ^1^ Canberra Rheumatology, Canberra, ACT, Australia; ^2^ OPAL Rheumatology, Sydney, NSW, Australia; ^3^ Monash University, Clayton, VIC, Australia; ^4^ Software4Specialists Pty Ltd, Sydney, NSW, Australia; ^5^ Barwon Rheumatology Service, Geelong, VIC, Australia; ^6^ Royal Melbourne Hospital, Parkville, VIC, Australia; ^7^ University of Sydney, Sydney, NSW, Australia; ^8^ Royal Prince Alfred, Camperdown, NSW, Australia; ^9^ Georgetown Arthritis, Newcastle, NSW, Australia

**Keywords:** patient reported outcomes, rheumatology, digitalisation, ePRO, patient—centered care, shared decision making

## Abstract

**Objective:**

To develop a simple and secure technological solution to incorporate electronic patient-reported outcomes (ePROs) into routine clinical care.

**Methods:**

A novel ePRO questionnaire delivery system was developed by Software for Specialists (S4S) in partnership with OPAL Rheumatology Australia. Validated questionnaires were sent from the electronic medical record (EMR) (Audit4) of patients with rheumatoid arthritis (RA), psoriatic arthritis (PsA), ankylosing spondylitis (AS), lupus or giant cell arteritis (GCA) and delivered to the patient's email address or completed in the clinic waiting room using a smart device (in-practice). Completed questionnaires were encrypted and returned to the patient's Audit4. Deidentified clinical data was extracted and aggregated across all sites. Data collected between April 2016-Dec 2020 were analysed descriptively.

**Results:**

Between April 2016 to Dec 2020, 221,352 Functional Assessment of Chronic Illness Therapy Fatigue (FACIT-F), Patient Health Questionnaire-2 (PHQ-2) and/or HealthCare Resource Utilization (HCRU) questionnaires were sent from 39 of 42 contributing clinics (93%). 85% of questionnaires were delivered *via* email and 15% in-practice. Overall, 85% of patients completed at least one questionnaire, and of all questionnaires sent, 73% were completed. Females were more likely to engage with the questionnaires than males (87% vs. 81%), and older patients were slightly more likely to complete all questionnaires delivered.

**Conclusions:**

The novel Audit4 ePRO delivery system is an effective tool for incorporating PROs into routine clinical care. The data generated provides a unique opportunity to understand the full burden of disease for patients in the real-world setting and the impact of interventions.

## Introduction

Reducing clinical disease activity to very low levels or remission for inflammatory arthropathies such as rheumatoid arthritis (RA), psoriatic arthritis (PsA) and ankylosing spondylitis (AS) is now possible with the availability of highly effective biologic and targeted synthetic disease modifying anti-rheumatic drugs (b/tsDMARDs) (1–3). Despite clinical remission being an achievable treatment target, subjective symptoms experienced by the patient which can be difficult to detect and measure objectively, can remain, including pain, fatigue, sleep disruption, depression, anxiety, social role participation and employment limitations (4). Patient reported outcomes (PROs) allow the patient to document how the disease impacts their quality of life in a standardised manner without being filtered through the lens of the clinician, providing additional context to measures of disease activity used by clinicians to make treatment decisions.

Registry studies and clinical trials are increasingly incorporating PROs to measure the full burden of disease and better understand the effectiveness and value of medicines (5, 6). The American College of Rheumatology (ACR), European League Against Rheumatism (EULAR) and Outcome Measures in Rheumatology (OMERACT) all recommend and endorse sets of outcome measures which include PROs for rheumatic diseases (7–9). When incorporated into routine care, PROs can help the clinician to better understand the patient experience, enhance communication and encourage shared decision making, which can empower the patient in the management of their own condition (10–12). However, the realities of time pressures during routine clinical care can significantly hamper recording of PROs in a regular or systematic fashion. Paper-based surveys can be burdensome for administrative staff, and despite tremendous innovation in digital health technologies and an abundance of applications harvesting consumer health data, there remains a lack of standardised digital methods for integrating PROs into the clinical workflow and records which does not rely on third parties to shuttle sensitive information between patient and clinician (13).

Further compounding these challenges, 80% of rheumatologists in Australia are practising in small independent community clinics without an overarching administrative structure so there is limited capacity for clinicians to develop and implement innovative clinical tools to enhance care at a scale that is economical. In 2009, the Optimising Patient outcomes in Australian rheumatology (OPAL)-Quality Use of Medicines Initiative (QUMI) was established to collate clinical data from rheumatologists distributed around Australia to allow large scale outcomes research to be conducted within the unique Australian rheumatology setting. A bespoke electronic medical record (EMR, Audit4) was customised for rheumatologists contributing to OPAL in partnership with software company Software4Specialists Pty Ltd (S4S) to capture comprehensive clinical data at the point-of-care which could be regularly deidentified, extracted and aggregated across all sites to create the OPAL Dataset. This EMR is now used by 111 rheumatologists (approximately 1 in 3) around Australia, predominately in private clinics (14).

In 2015, OPAL and S4S developed technology to seamlessly integrate electronic PROs (ePRO) into the routine consultation by delivering questionnaires to the patient through the EMR, and completed questionnaires are returned securely to the patient's EMR for review at the next consultation. This paper briefly describes real-world patient engagement with this technology by assessing the demographics and completion rates of Functional assessment of Chronic illness Therapy Fatigue (FACIT-F), Patient Health Questionnaire-2 (PHQ-2), and Health Care Resource Utilization (HCRU) questionnaires delivered using this technology as part of routine clinical care.

## Methods

### Data source

**Figure 1:**
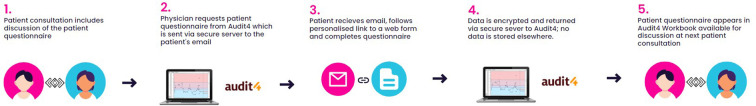
Electronic patient reported outcome delivery system workflow in Audit4.

A novel ePRO questionnaire delivery system was developed by Software4Specialists Pty Ltd (S4S) in partnership with OPAL Rheumatology. Validated PRO questionnaires were sent from the patient's EMR (Audit4, Software4Specialists Pty Ltd, Sydney, Australia) and delivered to the patient's email address at time intervals specified by the rheumatologist (defaults to quarterly) or completed in the clinic waiting room prior to the consultation using a tablet or the patient's smart phone (in-practice). Both options of delivery (email or in-practice) were available, and the rheumatologists could choose the delivery method most suitable for them and their patient. Completed questionnaires were encrypted and returned directly to the patient's Audit4 EMR held on the clinician's server for review at the next clinical consultation (Figure 1). The link to the PRO questionnaire expired within 28 days if the questionnaire was not completed, and the questionnaires were automatically cancelled if two consecutive links expired. This technology was made available to up to 111 rheumatologists located in 42 clinics in 6 states/territories in Australia (New South Wales, Queensland, Victoria, Western Australia, Tasmania and Australian Capitol Territory), and the use of this technology to furnish the clinical consultation was voluntary for clinicians and patients. Deidentified clinical data was extracted from the servers of participating rheumatologists and aggregated across all sites.

### Patient population

Sending of ePRO questionnaires was at the discretion of the treating rheumatologist in consultation with the patient and completion of the questionnaire by the patient was voluntary. All adult patients with a physician diagnosis of RA, AS, PsA lupus or GCA were included in the analysis if they were sent at least one FACIT-F, HCRU or PHQ-2 questionnaire between April 2016–December 2020. Patient demographics (age, gender) and diagnosis were analysed descriptively against the status (completed, expired, cancelled) of each questionnaire activated.

### Patient-reported outcomes

This study focused on describing the demographics and completion rates of three questionnaires, the FACIT-F, PHQ-2 and the HCRU. The FACIT-fatigue is a validated, commonly used, 13-item measure of fatigue. Questions are scored on a 0–4 response scale, ranging from “Not at all” to “Very much so”. The over-all fatigue score can range between 0 and 52 with higher scores representing less fatigue (15, 16). The questions in the FACIT-F questionnaire can be viewed at https://www.facit.org/measures/FACIT-Fatigue.

The PHQ-2 is a brief, two-question questionnaire adapted from the PHQ-9. It is a validated measure, intended to be used as an initial screening for major depression. The PHQ-2 asks the patient about the frequency of depressed mood and anhedonia over the past two weeks and identifies individuals who require additional evaluation. It does not determine whether a patient meets the criteria for a depressive order (17–19). The questions included in the PHQ-2 can be seen in Supplementary Figure S1.

The HCRU questionnaire asked about medical events and treatments experienced by the patient in the 3 months prior. The questions focused on visits to doctors or nurses, emergency department visits, hospitalisations, outpatient procedures, visits to allied health practitioners and alternative health practitioners (questionnaire can be viewed in Supplementary Figure S2).

The activities of OPAL Rheumatology Ltd have received overarching ethics approval from the University of New South Wales (UNSW) Human Research Ethics Committee (HREC), based on a patient opt-out arrangement (HC17799). All analyses were performed using R version 4.0.2.

## Results

Between April 2016-Dec 2020, 221,352 questionnaires (FACIT-F, PHQ-2 and/or HCRU) were sent from 39 of 42 contributing clinics (93%) to patients with a physician diagnosis of RA, AS, PsA, lupus or giant cell arteritis (GCA) (Table 1). The gender and age categories of patients receiving PROs were broadly representative of the OPAL cohort with females and patients 61–70 years old receiving the highest percentage of the total PROs sent. The percentage of PROs sent to patients with RA, AS and PsA was proportionally higher than the number of patients with these conditions in the OPAL dataset (Table 1).

**Table 1:** Demographic and clinical features of patients in the total OPAL cohort and patients in each PRO dataset.

	OPAL cohort (*n* = 203,593)	FACIT-F (*n* = 5,644)	PHQ-2 (*n* = 5,297)	HCRU (*n* = 5,230)
**Gender**				
Female, *n* (%)	135,881 (67.8%)	3,858 (68.4%)	3,726 (70.4%)	3,679 (70.4%)
Male, *n* (%)	63,234 (31.6%)	1,762 (31.2%)	1,547 (29.2%))	1,529 (29.3%)
Unassigned, *n* (%)	1,230 (0.6%)	19 (0.3%)	20 (0.4%)	18 (0.3%)
**Age**				
18–30, *n* (%)	10,711 (5.3%)	360 (6.4%)	304 (5.7%)	297 (5.7%)
31–40, *n* (%)	18,540 (9.2%)	624 (11.1%)	520 (9.8%)	509 (9.7%)
41–50, *n* (%)	27,049 (13.4%)	974 (17.3%)	841 (15.9%)	817 (15.6%)
51–60, *n* (%)	36,880 (18.3%)	1,297 (23.0%)	1,253 (23.7%)	1,235 (23.6%)
61–70, *n* (%)	43,654 (21.7%)	1,410 (25.0%)	1,384 (26.1%)	1,381 (26.4%)
71–80, *n* (%)	39,209 (19.5%)	840 (14.9%)	844 (15.9%)	843 (16.1%)
Over 80, *n* (%)	25,518 (12.7%)	138 (2.4%)	150 (2.8%)	147 (2.8%)
**Diagnoses**				
RA, *n* (%)	52,190 (25.6%)	3,748 (66.4%)	3,762 (71.0%)	3,726 (71.2%)
PsA, *n* (%)	15,365 (7.5%)	1,066 (18.9%)	1,056 (19.9%)	1,039 (19.9%)
AS, *n* (%)	5,634 (2.8%)	818 (14.5%)	446 (8.4%)	438 (8.4%)
Lupus, *n* (%)	4,250 (2.1%)	150 (2.7%)	153 (2.9%)	149 (2.8%)
GCA, *n* (%)	1,694 (0.8%)	19 (0.3%)	22 (0.4%)	22 (0.4%)

The majority (85%) of FACIT-F, HCRU and PHQ-2 questionnaires were delivered to the patient *via* email and 15% were completed in-practice. Overall, 85% of patients completed at least one FACIT-F, PHQ-2 or HCRU questionnaire (Table 2). 73% of all FACIT-F, PHQ-2 or HCRU questionnaires sent were completed. Although high completion rates were seen for both methods of delivery, worksheets done in practice had significantly higher completion rates than those delivered *via* email (Table 2). Importantly, these high completion rates were sustained over time with patients completing multiple PRO questionnaires. The median [inter-quartile range, (IQR)] number of questionnaires completed per patient was 3 (1,7) and the median (IQR) time since the first questionnaire was completed was 13 months (5,26) (Table 2).

**Table 2:** Features of worksheets in each PRO dataset.

	FACIT-F (*n* = 34,427)	PHQ-2 (*n* = 32,729)	HCRU (*n* = 32,349)
Completion rate	72.9%	73.0%	72.5%
Engagement rate^a^	84.8%	84.8%	84.6%
**Method of sending worksheet**			
In practice, *n* (%)	5,169 (15.0%)	5,068 (15.5%)	5,053 (15.6%)
By email *n*, (%)	29,210 (85.0%)	27,623 (84.5%)	27,248 (84.4%)
**Completion rate for method**			
In practice, *n* (%)	96.2%	94.60%	94.4%
By email, *n* (%)	69.0%	69.2%	68.7%
Number of completed worksheets per patient, median (IQR)	3 (1, 7)	3 (1, 7)	3 (1, 7)
Months of follow-up since first worksheet, median (IQR)	13 (5, 26)	13 (6, 26)	13 (6, 26)
Worksheet completion rate over time, median (IQR)	80.6% (77.0%, 83.5%)	80.4% (76.3%, 84.0%)	79.8% (75.8%, 82.6%)

^a^ Patients completing at least 1 worksheet.

The longitudinal completion pattern for patients in each PRO dataset is shown in Table 3. Males were slightly more likely to not complete any of their FACIT-F, PHQ-2 or HCRU questionnaires (<20%) compared to females (<14%), while older individuals (over 80 years of age) were slightly more likely to complete all questionnaires (≥50%) compared to those 70 years and under.

**Table 3:** Longitudinal completion pattern for patients in each PRO dataset.

	FACIT-F (*n* = 5,644)			PHQ-2 (*n* = 5,297)			HCRU (*n* = 5,230)		
Completion pattern^a^	All (*n* = 1,855, 33.0%)	Some (*n* = 2,913, 51.8%)	None (*n* = 854, 15.2%)	All (*n* = 1,722, 32.6%)	Some (*n* = 2,759, 52.2%)	None (*n* = 802, 15.2%)	All (*n* = 1,678, 32.2%)	Some (*n* = 2,736, 52.5%)	None (*n* = 802, 15.4%)
**Gender** **^b^**									
Female, *n* (%)	1,296 (33.7%)	2,033 (52.9%)	517 (13.4%)	1,244 (33.5%)	1,965 (52.9%)	508 (13.7%)	1,220 (33.6%)	1,944 (53.0%)	506 (13.8%)
Male, *n* (%)	556 (31.7%)	863 (49.2%)	334 (19.1%)	476 (30.8%)	777 (50.4%)	290 (18.8%)	456 (29.9%)	776 (50.9%)	293 (19.2%)
Unassigned, *n* (%)	3 (15.8%)	14 (73.7%)	2 (10.5%)	2 (10.0%)	15 (75.0%)	3 (15.0%)	2 (11.1%)	14 (77.8%)	2 (11.1%)
**Age** **^c^**									
18–30, *n* (%)	127 (36.1%)	160 (45.5%)	65 (18.5%)	105 (35.5%)	141 (47.6%)	50 (16.9%)	102 (35.3%)	140 (48.4%)	47 (16.3%)
31–40, *n* (%)	180 (29.0%)	324 (52.24%)	117 (18.8)	149 (28.7%)	274 (52.7%)	97 (18.7%)	143 (28.1%)	269 (52.8%)	97 (19.1%)
41–50, *n* (%)	283 (29.2%)	522 (53.8%)	165 (17.0%)	230 (27.4%)	464 (55.2%)	146 (17.4%)	220 (27.0%)	451 (55.3%)	145 (17.8%)
51–60, *n* (%)	408 (31.6%)	687 (53.1%)	198 (15.3%)	390 (31.2%)	663 (53.1%)	196 (15.7%)	373 (30.3%)	661 (53.7%)	197 (16.0%)
61–70, *n* (%)	475 (33.7%)	746 (53.0%)	187 (13.3%)	445 (32.2%)	746 (53.9%)	193 (13.9%)	451 (32.7%)	739 (53.5%)	191 (13.8%)
71–80, *n* (%)	313 (37.3%)	423 (50.4%)	104 (12.4%)	326 (38.6%)	417 (49.4%)	101 (12.0%)	316 (37.5%)	421 (49.9%)	106 (12.6%)
Over 80, *n* (%)	69 (50.0%)	51 (37.0%)	18 (13.0%)	77 (51.3%)	54 (36.0%)	19 (12.7%)	73 (49.7%)	55 (37.4%)	19 (12.9%)
**Completion method** **^d^**									
All by email, *n* (%)	957 (21.2%)	2,745 (60.9%)	809 (17.9%)	769 (4.0%)	2,555 (63.2%)	720 (17.8%)	731 (18.4%)	2,527 (63.5%)	721 (18.1%)
All in practice, *n* (%)	873 (83.9%)	123 (11.8%)	44 (4.2%)	920 (66.7%)	149 (13.0%)	77 (6.7%)	916 (79.9%)	154 (13.4%)	76 (6.6%)
Combination, *n* (%)	25 (35.2%)	45 (63.4%)	1 (1.4%)	33 (22.1%)	55 (59.1%)	5 (5.4%)	31 (34.1%)	55 (60.4%)	5 (5.5%)

^a^ Each patient categorised according to their worksheet completion pattern, with “All” consisting of those completing 100% of worksheets, “Some” consisting of those completing 1–99% of their worksheets and “None” consisting of those completing 0% of their worksheets.

^b^ Percentages calculated within each gender category.

^c^ Percentages calculated within each age category.

^d^ Each patient categorised according to their pattern of worksheet completion method, with “Combination” consisting of those who completed worksheets both by email and in practice.

When the completion method for patients in each PRO dataset was assessed longitudinally, most age groups (80 years and under) were found to be more likely to complete all FACIT-F, PHQ-2 or HCRU questionnaires that were sent by email, although individuals 80 years and older, were more likely to complete all in practice. No meaningful differences in the completion methods were seen between males and females (Table 4).

**Table 4:** Longitudinal completion method for patients in each PRO dataset.

	FACIT-F (*n* = 5,644)			PHQ-2 (*n* = 5,297)			HCRU (*n* = 5,230)		
Completion method^a^	All by email (*n* = 4,507, 79.9%)	All in practice (*n* = 1,040, 18.4%)	Combination (*n* = 17, 1.3%)	All by email (*n* = 4,041, 76.3%)	All in practice (*n* = 1,146, 21.6%)	Combination (*n* = 93, 1.8%)	All by email (*n* = 3,976, 76.0%)	All in practice (*n* = 1,146, 21.9%)	Combination (*n* = 91, 1.7%)
**Gender** **^b^**									
Female, *n* (%)	3,020 (78.5%)	784 (20.4%)	42 (1.1%)	2,797 (75.2%)	853 (22.9%)	67 (1.8%)	2,753 (75.0%)	852 (23.2%)	65 (1.8%)
Male, *n* (%)	1,468 (83.7%)	256 (14.6%)	29 (1.7%)	1,224 (79.3%)	293 (19.0%)	26 (1.7%)	1,205 (79.0%)	294 (19.3%)	26 (1.7%)
Unassigned, *n* (%)	19 (100.0%)	0 (0.0%)	0 (0.0%)	20 (100.0%)	0 (0.0%)	0 (0.0%)	18 (100.0%)	0 (0.0%)	0 (0.0%)
**Age** **^c^**									
18–30, *n* (%)	266 (75.6%)	83 (23.6%)	3 (0.9%)	209 (70.6%)	85 (28.7%)	2 (0.7%)	202 (69.9%)	84 (29.1%)	3 (1.0%)
31–40, *n* (%)	528 (85.0%)	86 (13.8%)	7 (1.1%)	423 (81.3%)	92 (17.7%)	5 (1.0%)	412 (80.9%)	94 (18.5%)	3 (0.6%)
41–50, *n* (%)	823 (84.8%)	135 (13.9%)	12 (1.2%)	694 (82.6%)	137 (16.3%)	9 (1.1%)	672 (82.4%)	134 (16.4%)	10 (1.2%)
51–60, *n* (%)	1,083 (83.8%)	195 (15.1%)	15 (1.2%)	1,011 (80.9%)	216 (17.3%)	22 (1.8%)	995 (80.8%)	216 (17.5%)	20 (1.6%)
61–70, *n* (%)	1,132 (80.4%)	251 (17.8%)	25 (1.8%)	1,069 (77.2%)	284 (20.5%)	31 (2.2%)	1,064 (77.0%)	283 (20.5%)	34 (2.5%)
71–80, *n* (%)	619 (73.7%)	215 (25.6%)	6 (0.7%)	579 (68.6%)	245 (29.0%)	20 (2.4%)	577 (68.4%)	249 (29.5%)	17 (2.0%)
Over 80, *n* (%)	60 (43.5%)	75 (54.3%)	3 (2.2%)	59 (39.3%)	87 (58.0%)	4 (2.7%)	57 (38.8%)	86 (58.5%)	4 (2.7%)

^a^ Each patient categorised according to their pattern of worksheet completion method, with “Combination” consisting of those who completed worksheets both by email and in practice.

^b^ Percentages calculated within each gender category.

^c^ Percentages calculated within each age category.

## Discussion

The data presented here reflects the real-world use of ePROs in routine clinical practice. The results demonstrate high engagement and completion rates of ePROs by patients with common rheumatological diagnoses across all age categories. Importantly, this high completion rate was also sustained over time and many patients had completed multiple ePRO questionnaires. This is important as it allows the treating rheumatologist to track their patient's PRO measures longitudinally and monitor improvement. The high engagement rates reported here are not surprising considering the positive effect PROs reportedly have on the patient-clinician relationship. In a qualitative study investigating perspectives of patients with RA on PRO data collection, patients expressed their interest in PRO measures and desire for greater involvement in the management of their disease (20). In addition, in a recent study describing the impact of integrating PROs into routine clinic, patients reported that PROs improved communication and shared decision making with their rheumatologist and made the visit more patient-centred. This resulted in high satisfaction and confidence in their treatment (12).

The development of an ePRO system that can be emailed to the patient for them to complete at home in their own time is a major advantage of the Audit4 software. A study that investigated patients preference for PRO collection found that patients prefer entering ePRO data at home (21). This remote access also enabled continued collection of ePROs during the coronavirus (COVID-19) pandemic.

The rise of technology to facilitate healthcare holds great potential to improve patient outcomes in an efficient and patient-centered manner. Despite the shift towards digital health, ePROs have not been widely implemented in routine clinical care. In a recent study conducted in Germany, the main reasons cited by rheumatologists for not implementing ePROs was the absence of suitable software, and the difficulties and high costs associated with implementing innovative software solutions (22). As such very few healthcare systems have built the analytic capacity to fully leverage the potential of PROs. The Audit4 EMR ePRO system has overcome these hurdles and to the best of our knowledge is the first of its kind to allow seamless integration of PROs into the daily workflow of a large network of rheumatologists based nationwide across Australia. This solution not only supports real-time decisions made by clinicians and patients but also incorporates a successful mechanism for collecting quantitative data that opens the potential for these important data to contribute to EMR-based research, facilitating representation of the patient perspective.

## Limitations

The decision to utilise the ePRO technology, and which patients they were sent to, was at the discretion of the rheumatologist resulting in possible selection bias which was not addressed in the current study. In addition, self -reported questionnaires are at risk of misinterpretation and bias due to inaccurate recall and social desirability which may impact the validity of results (23). For this reason, it is important to use validated PRO measures to better ensure the questionnaires are interpretable, reliable and measure what they claim to measure.

## Future work

Due to the successful integration and real-world utilisation of the Audit4 ePRO system, important patient reported quality of life data is being captured within the OPAL dataset and available for future research questions. A follow-on study would be to describe the levels of fatigue, mood disturbance and healthcare resource utilisation in our cohort of RA, AS, PsA GCA and lupus patients. The relationship between PRO measures and the traditional clinical disease activity measures as well as investigating the impact ePROs have on patient outcomes are also questions needing further investigation.

## Data Availability

The data that support this study cannot be publicly shared and may be shared upon reasonable request to the corresponding author if appropriate. Requests to access the datasets should be directed to info@opalrheumatology.com.au.
