# Supplementary material for: A novel electronic patient-reported outcome delivery system to implement health-related quality of life measures in routine clinical care: An analysis of 5 years of experience
Source: Front Digit Health. 2023 Jan 9;4:1074931. doi: 10.3389/fdgth.2022.1074931 (PMC9869675; doi:10.3389/fdgth.2022.1074931)
Supplement: Supplementary file 1 [file Datasheet1.docx]

**A novel electronic patient-reported outcome delivery system to implement health-related quality of life measures in routine clinical care: An analysis of 5 years of experience.**

**Supplementary Files**

# Supplementary Figure 1. Patient Health Questionnaire-2 (PHQ-2)

# Supplementary Figure 2. Healthcare Resource Utilisation Questionnaire (HRCU)

#
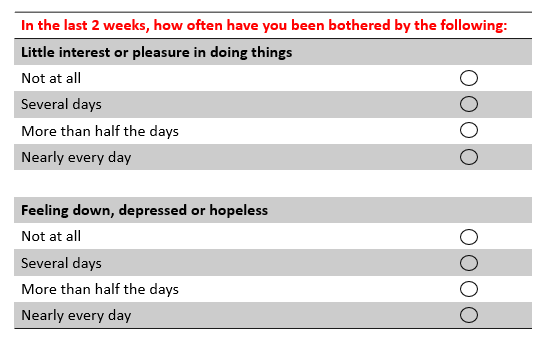
Supplementary Figure 1. Patient Health Questionnaire-2 (PHQ-2)


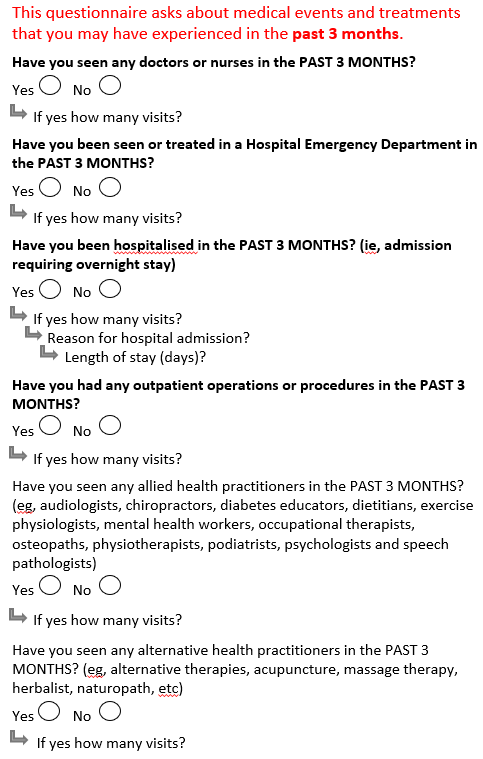


# Supplementary Figure 2. Healthcare Resource Utilisation Questionnaire (HRCU)
